# J Guy Edwards, FRCPsych, FRCP, FRCGP (Hon), DPM, HonMFPH, Founding Editor of *Human Psychopharmacology*

**DOI:** 10.1002/hup.2860

**Published:** 2022-11-30

**Authors:** Philip Cowen

**Affiliations:** ^1^ Department of Psychiatry University of Oxford Oxford UK

**Keywords:** psychopharmacology

1

Guy Edwards dedicated his life to the clinical care of patients and was a full‐time NHS consultant psychiatrist in Southampton until his retirement in 1993. This made his numerous achievements in psychopharmacology ‐ carried out of necessity on top of his NHS work in his own time—particularly remarkable. In fact, coming from a working‐class family in a Welsh mining village, Guy was the first member of his family to have full experience of secondary school, let alone a university education.

Admiration for the family GP prompted Guy to opt for medicine, and positive experiences of work with psychiatric patients as a medical student led him later to specialise in psychiatry in Manchester where he obtained his DPM in 1964. However, it was a chance encounter with Linford Rees that led Guy into the field of psychopharmacology. Rees knew that the famous American psychopharmacologist, Nathan Kline, was in London recruiting psychiatrists to assist in his drug development programme and arranged a meeting between them.

The meeting with Kline resulted in Guy spending three key years in the United States where he learned about laboratory and clinical aspects of psychopharmacology, the latter in collaboration with George Simpson involving clinical trials of antipsychotic drugs in the ‘Early Clinical Drug Assessment Unit (ECDU)’ at the Rockland Research Institute, New York. This was an exciting time for psychotropic drug development with the antipsychotic effects of chlorpromazine having been demonstrated just a few years earlier. At ECDU, Guy studied the clinical effects of several newer potential antipsychotic drugs, including thioxanthenes and butyrophenones. The experience gained here was important to Guy, when after returning to the UK, he carried out investigations of the therapeutic and adverse effects of other new antipsychotic agents such as remoxipride and sulpiride. Characteristically in this work he would devise his own protocols and be responsible personally for patient recruitment and clinical care.

Guy's contribution to the psychopharmacology of antidepressant drug treatment was particularly notable and showed his gift for collaboration with general medical and psychiatrist colleagues as well as his ability to identify issues important to clinicians and their patients. For example, recognising the problematic pro‐convulsant effects of tricyclic antidepressants, Guy carried out studies with Michael Sedgwick, a Professor of Neurophysiology, looking at the effect on seizure threshold of alternative antidepressant agents such as mianserin and paroxetine. Similarly, with physician‐pharmacologist Derek Waller, he compared the effects of antidepressants on cardiac conduction and assessed the consequences of lithium treatment on renal function.

Guy's combination of scientific insight and clinical acumen led him to be a frequent editorial writer for journals such as the British Medical Journal (BMJ). His judgement here was always informed by available evidence and the welfare of patients and has stood the test of time well. For example, his conclusion for BMJ readers (BMJ 304, 1644‐5) that SSRIs represented a ‘modest though welcome advance’, in antidepressant drug therapy, though raising the ire of some in Industry, now seems quite correct. In fact, Guy's subsequent article (co‐authored with Ian Anderson), ‘Systematic review and guide to selection of selective serotonin reuptake inhibitors’ (Drugs, 1999, 57, 507–533) was probably his most widely read paper with over 350 citations in Google Scholar.

Guy was also an early advocate of placebo‐controlled trials of new antidepressant agents at a time when this approach was unfashionable and often rejected by ethics committees. Of course, it is now widely accepted that placebo‐controlled trials are necessary to demonstrate specific efficacy of new antidepressant treatments and that such studies have an important role in preventing the promulgation of ineffective therapies. Guy's summary of the ethical principles supporting placebo‐controlled trials of antidepressants and the way in which such studies can be carried out practically and safely in depressed patients still makes compelling reading (*Human* *Psychopharmacology,* 1989, 4, 235–236).

Subsequently, Guy formed a fruitful collaboration with Bill Inman, a Professor of Pharmaco‐epidemiology and founder of the Southampton Drug Safety Research Unit (DSRU). Their work, using prescription event monitoring of adverse effects in primary care, led to influential papers looking at the harms of widely used psychotropic drugs such as alprazolam, fluvoxamine and fluoxetine. Guy's knowledge of the increasingly important area of drug safety led to him being invited to contribute articles to numerous journals such as the Drugs and Therapeutic Bulletin, Prescribers Journal and CNS Drugs as well as several psychopharmacology textbooks including those published by the British Association for Psychopharmacology (BAP). In fact, Guy was a member of the BAP from its inception and was Honorary Treasurer from 1984 to 1989. During this period the investment income of the organisation flourished but, with typical modesty, Guy was quick to attribute this to the idiosyncrasies of the market rather than any Warren Buffett‐like financial acumen on his part.

One of Guy's most lasting contribution to the psychopharmacology field will be his work as Founder Editor‐in‐Chief of a new journal, ‘*Human Psychopharmacology*’. Guy spent 18 months working on the aims of the journal and developing the team of co‐editors and international editorial advisory board before the first issue was published in September 1986. The journal flourished but after eight years Guy found that in the context of his full time NHS post, the burden of editorship had too substantial an impact on his own writing and research as well as other commitments (*Human Psychopharmacology* 8, 379–380). However, Guy continued his close relationship with the journal and was able subsequently to co‐author an editorial which described 25 successful years of publication as well as welcoming the current editor, David Baldwin (*Human Psychopharmacology,* 2012, 27, 1–3).

Psychopharmacology continues to be a treatment modality through which psychiatrists can offer real benefits to their patients; however, very few full‐time NHS consultants have the energy and vision to produce the kind of contribution that Guy Edwards made to clinical psychopharmacology over 30 years of practice. Hopefully, in the future, more effective, pharmacological approaches will become available but whatever the therapeutic advances, Guy's example of a practitioner dedicated to the clinical science and practical application of psychopharmacology for the well‐being of their patients will not be bettered.

